# Cohesive Tin

**Published:** 1904-08

**Authors:** W. M. Randall

**Affiliations:** Professor of Operative Technics, Dental Anatomy and Comparative Dental Anatomy in the Louisville College of Dentistry.


### COHESIVE TIN.

By W. M. Randall, D. D. S.

Professor of Operative Technics, Dental Anatomy and Comparative Dental Anatomy in the Louisville College of Dentistry.

(Written for *The American Journal of Dental Science.*)

The exceptional qualities of tin as applied to operative dentistry have long been recognized by the experienced operators of the profession as a potent element in the successful results attained by the so called "Stopping Process."

Its highest standard of softness, at the same time retaining its toughness and strength, renders it easy of manipulation, more uniform and certain in its adaptability than the Noble Metals of gold and platinum. The *reciprocal* relation of tin and gold—in combination—will prevent the active conduction of thermal changes and also give a preserving property unequalled by any other of the filling materials either in combination or independently.

The practical daily use of *Cohesive-tin* in the operative field is restricted to comparatively few men in the profession. Still if its *really meritorious* properties and indications were more widely known among our own practitioners, there would be very few dentists who would not religiously consider it one of the reliable factors necessary to a safe and successful practice. The term *cohesion* is very prone to be mis-applied in the accurate designation of tin-foils as placed upon the market under the general head of "dental supplies." This most *vital* and important physical property of tin can only be fully appreciated in the use of "Chemically Pure" and non-contaminated metal. And we might add, can not be found except in the bar of pure tin ore with exception in one or two of the tin-foils as found in the dental supply houses, newly manufactured and *especially* treated for non-contamination by gases.

*Indications.*—Cohesive-tin can be used to great advantage on unexposed surfaces of the bicuspids or molars; although it may be used on the lingual surfaces, and the *interior* of gold fillings on the anterior teeth. It does not permeate the tooth structure as the amalgams and will not give a darkened effect of the enamel or in the least discolor, except in some mouths, the immediate area of the exposed surface of the tin may discolor.

The writer prefers in the *preparation* of *Cohesive-tin* to cut from a fresh surface of pure bar-tin with an ordinary, clean bladed pocket knife and by a steady and forcible scraping motion cut long thin and regular shavings from the bar, thus readily and conveniently preparing the material to suit the operator and the size of the cavity at hand. The cavity should also be exempt from all signs of moisture and properly prepared as for cohesive-gold—although the point angles need not be so accurate. The cavity preparation and cavity toilet should be ready for the immediate introduction of the *freshly* cut tin.

Presuming a complex disto-occlusal single-step cavity on the inferior first or second bicuspid prepared for the reception of the filling;—the operation of filling in order of procedure would be as follows: viz:—(a) Apply a closely fitting matrix, carefully observing all *lines* of contour for the resultent filling and accurately force a wooden wedge in the inter-proximate space until you get perfect adaptation of matrix against the gingival margin of the cavity. (b) Using serrated gold pluggers proceed to introduce the long tin shavings in a similar manner as for cohesive-gold ropes. However greater quantities of the tin may be condensed under the plugger than of gold consequently, it is more rapid in the filling process. (c) Care should be exercised to see that the tin is sufficiently condensed throughout to secure perfect adaptation to the walls of cavity and matrix; but its *vital* working properties may be seriously injured by heavy and continued condensation. (d) The tin should not be brought nearer the occlusal surface than the gingival third of cavity and in cases where it would be exposed to direct view it may be veneered with cohesive-gold foil even in those conditions. (e) There should be enough separate retention in the walls of the cavity yet unfilled to hold the cohesive-gold in the cavity, independently of any cohesion it may have with the tin;—although its strong adhesion, due to gummy surface of the freshly cut tin and the inherent strength of the mechanical interdigitation produced by the twisting motion of condensing the gold on surface of tin with serrated pluggers—seems to give the effect of cohesion, but it is not complete thus it is not true cohesion; as will be proven by the fact that it will flake or peel off of the tin unless this feature of separate retention is duly considered. (f) Owing to the softness of the entire mass of tin the matrix should not be removed until all the condensing force has been applied to the filling, without the support of the matrix the tin base may slightly flow, but with the hard surface of the gold exposed to the stress of mastication after the filling is finished this feature is removed. (g) The finishing process is made very easy and simple, especially at that most vulnerable point just below the gum gingiva. The excess may be removed with the use of strong linen or light emery or sand-paper strips about two millimeters wide; and the perfect adaptation due to the softer quality peculiar to tin for tooth structure, makes the gingival third of the filling much less liable to the ingress of secondary decay.

Cohesive-tin is highly indicated in contour fillings on the six anterior teeth where the cavity is deep and there is probability of pulp-irritation and finally hyperemia as a result of thermal change. The mere *presence* materially reduces and in many instances entirely prevents the uncomfortable or irritating effects of hot or cold.

*Reflections.*—Use "chemically pure" bar tin.

Cohesively the use of tin is principally in contour fillings in combination with cohesive-gold.

The material should be used freshly cut from the bar, and not allowed to become oxidized or in any way contaminated before using.

It should not be introduced in the cavity in such a manner as to be exposed to direct view when the filling is finished.

Always use an accurately fitting and strong matrix and let it remain in position until entire filling has been introduced.

When the tin is exposed at the gingival third of the cavity, it is more easily and perfectly finished than with any cohesive-gold. Adaptation in difficult positions may be had with much less effect to the operator and is more perfect than cohesive-gold.

Tin coheres to tin, and gold coheres to gold, thus perfectly welding:—Gold does not cohere to tin but the adhesion is electrically and mechanically perfect and besides excluding any carios action on the surrounding tissue the combination adds a peculiar preserving property to the finished filling.

The redeeming virtues of this combination can not be fully appreciated until tested in more than one subject. Give it a trial and be your own judge.

---

### PRESSURE ANAETSHESIA.

By Dr. G. B. Speer, Youngstown, O.

(Written for *The American Journal of Dental Science.*)

